# Immunosuppressive Potential of Activated Human Amniotic Cells in an Experimental Murine Model of Skin Allo- and Xenotransplantation

**DOI:** 10.3389/fmed.2021.715590

**Published:** 2021-09-23

**Authors:** Emanuel Kolanko, Aniela Grajoszek, Piotr Czekaj

**Affiliations:** ^1^Department of Cytophysiology, Chair of Histology and Embryology, Faculty of Medical Sciences in Katowice, Medical University of Silesia, Katowice, Poland; ^2^Department of Experimental Medicine, Medical University of Silesia in Katowice, Katowice, Poland

**Keywords:** human amniotic stem cells, skin transplantation, allografts, xenografts, cyclosporine A

## Abstract

Isolated human amniotic cells (hAC) could be used as a source of immunomodulatory factors in regenerative medicine and transplantation. However, in previous experimental studies, native hAC administered to skin graft recipients did not induce graft immunotolerance. To strengthen the immunomodulatory properties of hAC prior to administration to the recipient, we activated them *ex vivo* using pro-inflammatory cytokines. In this study, we compared the transplantation efficiency of skin allografts (mouse to mouse) and xnografts (rat to mouse) in recipient mice divided into three main groups receiving: 1. Placebo (control group); 2. Cyclosporine A (CsA) [10 or 50 mg/kg body weight (bw)]; 3. suspension of hAC activated *ex vivo* by IL-1β and INFγ, administered into a tail vein or subcutaneously. During 15 days of observation, hAC administered intravenously or subcutaneously after allotransplantation appeared to be as safe and efficient as CsA at the dose of 10 mg/kg bw in preventing rejection of skin allo- and xenografts. After xenotransplantation, however, only hAC administered intravenously prevented rejection to an extent comparable to CsA. Both CsA (10 mg/kg bw) and activated hAC reduced inflammatory infiltration in the skin (after intravenous injection) and did not increase the concentration of the inflammation marker SAP in serum or percentage of leukocytes in blood. Finally, we concluded that administration of activated hAC is safe and efficient in the presented animal model of skin allo- and xenotransplantation in a route-dependent manner. Activated hAC injected intravenously exhibit an immunosuppressive effect comparable to CsA administered at the dose of 10 mg/kg bw in both allo- and xenotransplantation.

## Introduction

Skin grafting, including auto-, allo-, and xenotransplantation, is currently an important clinical topic. Most often, these treatments are performed due to thermal damage to the patient's skin to replace areas of skin loss or to use skin as a kind of dressing. Such a dressing protects the body against excessive fluid loss, metabolic disorders, and infections (1). Owing to their complete immunological tolerance, autographs have the most advantages; however, due to insufficient biological material, it is necessary to use skin taken from another person or foreign species, most often from pig donors (2). Alternatively, instead of skin grafts, attempts are being made to use different types of bioproducts based on tissue gels and adhesives, which often include stem cells (3). In clinical practice, the success of skin allo- and xenotransplantation is related to, among others, postoperative immunosuppression, whose aim is to induce tolerance of the recipient's organism to foreign tissue. The most commonly used immunosuppressants include drugs acting on immunophilins, such as cyclosporin A (CsA) and tacrolimus (4, 5). Unfortunately, pharmacological immunosuppression causes many side effects, such as hypertension, hypercalcemia, hyperlipidemia, trophic changes in the gums, gout, and severe impairment of kidney, liver, and skin functions (6).

An alternative solution or supporting tool for conventional pharmacological immunosuppression could be stem cell therapies. One of the attractive sources of tissue stem cells is the human amniotic membrane due to, among others, unique immunomodulatory properties of amniotic cells and their ability to differentiate into three germ layers. Unlike other stem cell populations such as human embryonic stem cells (hESC) or induced pluripotent stem cells (iPSC), cells isolated from the amnion—human amniotic cells (hAC)—are readily available, their isolation is not ethically controversial, and after transplantation they do not form neoplastic tumors (7).

Despite many potential benefits of using hAC in cell therapies, there are still unresolved technical problems with their transplantation related to appropriate *ex vivo* preparation prior to administration to the recipient. Until now, experimental animal models of treatment of e.g. autoimmune diseases or a skin transplantation model (8) have mainly used native cells administered immediately after their thawing from the cell bank, without additional stimulation of their function by culturing them with appropriate growth factors or interleukins. Studies on populations of mesenchymal stem cells (MSC) suggest that this approach may limit their therapeutic potential (9). One solution could be the *in vitro* activation of stem cells. We have previously shown that the addition of some cytokines such as interleukin 1β (IL-1β) or interferon γ (INFγ) to the culture medium allows to increase the secretion of immunomodulatory substances by hAC with a maximum activation after 96 h of culture (10). Consequently, the cells prepared this way can be used as a more efficient source of immunosuppressants acting as an adjunct or alternative to conventional drug immunosuppression in skin allo- and xenotransplantation.

In this study we verified a hypothesis that immunosuppresory properties of amniotic cells enhanced by *ex vivo* activation are comparable to, or more pronounced, than that of Cyclosporine A in terms of sufficiency for inhibition of graft rejections.

The aim of this experimental study was a comparative assessment of the transplantation efficiency of the skin allo- (mouse-to-mouse) and xeno- (rat-to-mouse) grafts in terms of immunosuppression dependent on Cyclosporine A or activated hAC. We also assessed the effectiveness of hAC depending on a route of its administration (via blood vs. transdermal injections) with comparison to Cyclosporine A transdermal injections.

## Materials and Methods

**Figure 1:**
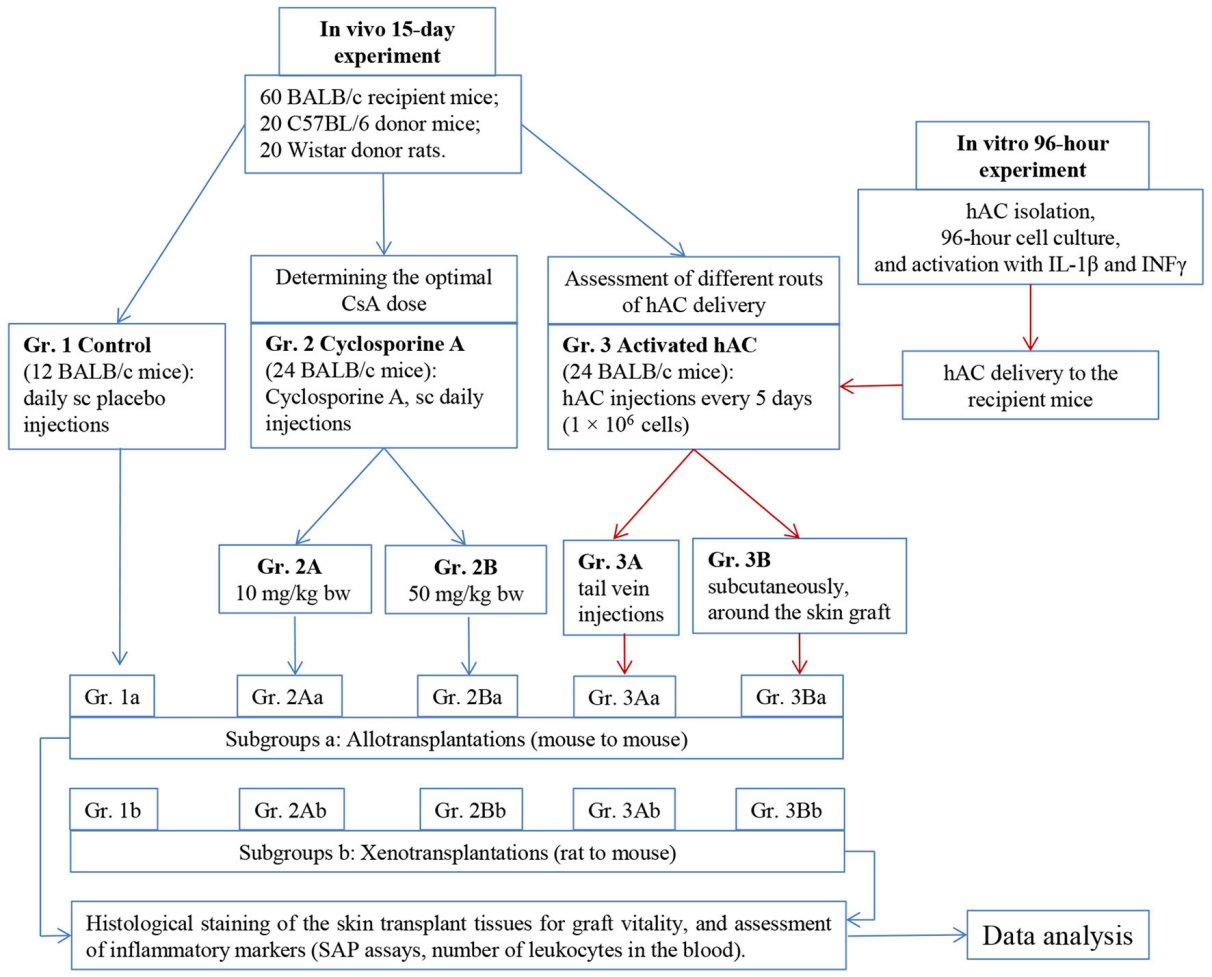
Outline of the work plan. Sc, subcutaneous injections; bw, body weight.

The experiment was performed using an allo- and xenotransplantation model in Balb/c mice (recipient mice) and C57BL/6 mice (donor mice) as well as Wistar rats (donor rats). The experiment used hAC activated *ex vivo* by IL-1β and INFγ, which were characterized at 12 and 96 h. We compared *in vivo* the immunosuppressive effects of IL-1β and INFγ-activated hAC and different doses of CsA. For this purpose, after skin allotransplantation (mouse to mouse) or xenotransplantation (rat to mouse), the recipient mice received the following injections: Group 1—placebo (control group), subcutaneously; Group 2—CsA, 10 (subgroup 2A) or 50 (subgroup 2B) mg/kg body weight (bw), subcutaneously; and Group 3—suspension of *ex vivo*-activated hAC (1 × 10^6^ cells), into the tail vein (subgroup 3A) or subcutaneously in the tissue surrounding the graft (subgroup 3B). Both, control group (gr. 1) and each experimental subgroup (gr. 2A, 3A and 2B, 3B) were subsequently subdivided into allo- (a) and xenotransplantation (b) groups. In each group, the recipient mouse observation lasted up to 15 days. During this time, the viability of the graft was examined visually and by palpation; the histological structure of the graft was also assessed. Markers of generalized inflammatory response in recipient mice were determined (Figure 1).

### Isolation and Activation of hAC

Amniotic cells were isolated from the placenta taken from a 26-year-old healthy woman after informed consent of the patient to collect the material for research and with the consent of the Bioethics Committee of the Medical University of Silesia (SUM) in Katowice (decision no KNW/0022/KB/229/14). The pregnancy was full term and uncomplicated, it was delivered at term by cesarean section for non-obstetric reasons (state after pelvic plastic surgery) in the absence of signs of placental insufficiency. The patient did not use stimulants, did not have non-obstetric chronic conditions, and had not been infected with HIV, HCV, HBV, or bacterial infections.

The placenta was collected into a sterile container containing phosphate buffered saline with the addition of an anticoagulant (5 mM of ethylenediaminetetraacetic acid, EDTA), antibiotic, and antimycotic (0.1 U/ml of penicillin, 0.1 mg/ml of streptomycin sulfate, and 0.25 μg/ml of amphotericin B). The collected placenta measured 22 × 23 cm and weighed 472 g, while the amniotic membrane extracted from the placental shield weighed 11 g. hAC cells were isolated by a series of enzymatic digestions: once with dispase (2.4 U/ml) for 7 min, twice with trypsin (0.05%) for 40 min per each digestion, and once with collagenase (0.75 mg/ml) for 60 min, obtaining 142 million hAC (10, 11). The cells were then activated by IL-1β and INFγ in a 96-h culture in TeSR2 medium according to a previously published protocol (11), and characterized at 12 and 96 h. Briefly, the activation efficiency was assessed by assays of *HLA-G, PTGS2*, and *iNOS* gene expression, HLA-G antigen surface expression, and soluble sHLA-G and PGE2 concentrations in the culture medium.

To determine *PTGS2, NOS2* and *HLA-G* gene expression RT-PCR arrays were performed. Total RNA was isolated from cultured hAC using the RNeasy Mini Kit (Qiagen). cDNA synthesis was performed using the RT2 First Strand Kit (Qiagen). Real Time PCR reaction was made using the RT2 qPCR-SYBR MasterMix and the RT2 Custom Profiler PCR Array System (Qiagen). The reference genes were PPIH and RPLP0. The reactions were completed in accordance with the manufacturers' protocol (RocheLightCycler480 apparatus). The results were analyzed using the Web-based PCR Array Data Analysis Software (pcrdataanalysis.sabiosciences.com/pcr/arrayanalysis.php).

hAC immunostaining was performed with anti-human primary antibodies for the expression of pluripotency marker SSEA4 [using anti-SSEA4 mouse monoclonal IgG3 (MC813)], epithelial marker CK7 [with anti-cytokeratin 7 mouse monoclonal IgG1 (OV-TL 12/30)], and HLA-G antigen [with anti-HLA-G mouse monoclonal IgG1 (MEM-G/9)]. Appropriate isotype controls were also used. Subsequently, the cells were incubated with secondary goat anti-mouse IgG (DyLight 488) antibody. The antibodies (all from Abcam) were used in accordance with manufacturers' protocol. Immunocytochemical reactions were analyzed under the fluorescence microscope Nikon Eclipse Ti-U, equipped with a camera (Nikon Digital Sight DS-SMc), running on the NISElements AR 3.00 (Nikon Instruments Inc.). To quantify expression of surface markers, hAC were resuspended and stained for SSEA4 and HLA-G markers and analyzed by flow cytometry (FACS Aria, BD) with specific antibodies: PerCP Mouse Anti-Human SSEA4 (R&D System) and PE Mouse Anti-Human HLA-G (eBioscience), and appropriate isotype controls. All the FACS analyses were performed using the BD FACSDiVa software and FlowJo software (LLC).

The concentrations of soluble HLA-G (HLA-G ELISA Kit; BioVendor) and PGE2 (PGE2 ELISA Kit; Invitrogen) were assessed in the supernatant obtaining after hAC cell culture centrifugation, using ELISA method in accordance with the manufacturers' protocol.

### Experimental Animals

Animal tests were carried out with the consent of the Local Ethical Committee on Animal Experiments (decision no 121/2015) of the SUM in Katowice.

The study used 60 eight-week-old female BALB/c mice, 20 eight-week-old male C57BL/6 mice, and 20 ten-week-old male Wistar rats obtained from the breeding facility of the Centre for Experimental Medicine of the SUM in Katowice. All animals were sexually mature. The starting weight of BALB/c mice averaged 22 g (± 2 g), C57BL/6 mice −23 g (± 2 g), and Wistar rats −347 g (± 35 g). During the experiment, the animals were kept in separate cages under standard conditions: a temperature of 22°C (± 2°C), humidity of 50–60%, light/dark cycle of 12/12 h, and light intensity of 60–400 lx. Water and food (Labofeed) were available *ad libitum*.

### Skin Grafts

The animal experiments involved transplantation of pieces of skin: BALB/c mice were transplanted with skin allografts obtained from C57BL/6 mice and xenografts obtained from Wistar rats. In the allograft group, full-thickness skin allografts measuring 1 cm × 1 cm obtained from eight-week-old C57BL/6 mice were transplanted onto the backs of eight-week-old BALB/c mice. In the xenograft groups, full-thickness skin specimens measuring 1 cm × 1 cm obtained from ten-week-old Wistar rats were transplanted onto the backs of eight-week-old BALB/c mice. From one donor animal, four pieces of skin were collected for transplantation and one skin piece for control histological examination. The skin specimens were taken from the donor animal under anesthesia (Propofol-Lipuro; B. Braun). Immediately after anesthesia, the animals were shaved at the site of the planned graft collection. The shaved area was disinfected. A full-thickness skin specimen was then cut out and placed in normal saline at a temperature of 4°C for several minutes. The graft donor animals were euthanized immediately after the skin specimens were taken.

At the same time, the animals in the recipient group were anesthetized (Propofol-Lipuro; B. Braun) and shaved in the area where the graft was to be placed. The area was disinfected and then the graft bed was prepared by cutting with scissors through the epidermis, dermis, and subcutaneous tissue up to the muscle layer. The grafts were placed in the beds, secured with several non-absorbable sutures, and covered with a dressing of sterile gauze and Leukoplast tape. The dressings were not removed for the first three days after the transplantation. After this time, the dressings were changed daily. Graft viability was examined visually and by palpation. Simultaneously, photographic documentation of the grafts was made using a camera (Nikon D3300). Complete graft rejection was defined as necrosis of the entire epidermis of the transplanted piece of skin. Each mouse with identified extensive but incomplete graft necrosis (about 80% of the area) was euthanized, and then a piece of the grafted skin and a piece of the surrounding skin of the recipient as well as blood were collected for further examination.

### Injections

#### Administration of CsA

CsA concentrate for intravenous infusion (0.05 g/ml; Sandimmun, Novartis Pharma GmbH, Germany) was dissolved in Kolliphor EL formulation (Sigma-Aldrich) at a concentration of 1 mg/100 μl and administered subcutaneously in the dorsal region of the recipient animal at a dose of 10 mg/kg or 50 mg/kg bw every 24 h. The amount of medication administered was determined each time prior to administration, after weighing the mouse.

#### Administration of Activated hAC

Amniotic cells, isolated and then activated with IL-1β and INFγ in a 96-h culture, were divided into aliquots of 1 × 10^6^ cells each, suspended in 100 μl normal saline, and administered to the recipient mice via the tail vein 15 min before skin grafting or subcutaneously in the region of the graft immediately after grafting. The cells were administered every 5 days using an insulin syringe with a 27-G, 0.5-inch (0.4 × 14) needle (Medical-Łomza).

#### Placebo Administration

The mice in the control group were administered 100 μl normal saline subcutaneously at a frequency corresponding to CsA injections.

### Assessment of Inflammatory Markers

The concentration of the specific inflammatory marker serum amyloid P component (SAP) in the blood plasma of the tested mice taken prior to euthanasia was determined by ELISA using Mouse SAP/PTX2 ELISA Kit (OriGene), according to the protocol attached by the manufacturer. The results were read on a Wallac Victor2 reader.

A microscopic analysis was performed of blood smears stained by the May-Grünwald-Giemsa method that assessed the percentage of individual morphotic elements of blood.

### Macroscopic Evaluation of the Degree of Graft Rejection

A subjective survival rate was used to macroscopically assess the rate of skin graft rejection. The degree of graft rejection was reported as the percentage of the graft area where skin necrosis was observed in relation to the total graft area (Table 1). Any additional skin lesions were excluded from the analysis.

**Table 1:** Percentage scale to characterize mouse skin graft rejection.

Day 0 − 0 points	Day 4 − 1 point First signs of graft rejection	Day 9 − 2 points Necrosis 20–49%	Day 11 − 3 points Necrosis 50–80%	Day 14 − 4 points Necrosis > 80%
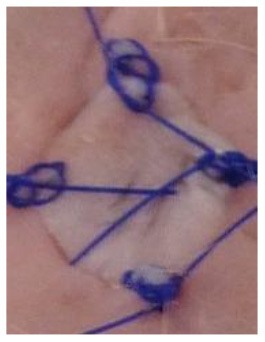	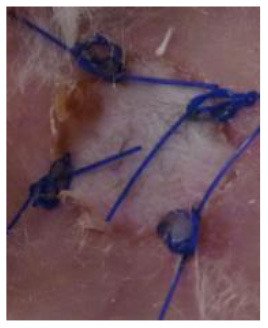	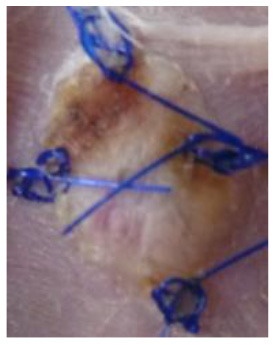	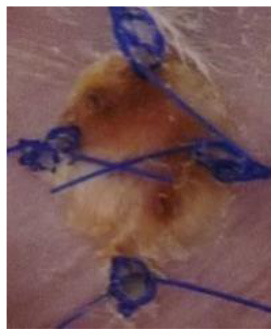	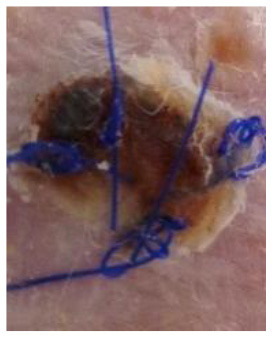

*This scale was essential for macroscopic evaluation of the graft vitality during the experiment. Different scores were used to define graft morphology, from intact graft, through early stages of rejection, up to massive graft rejection (necrosis >80% of the graft area)*.

### Microscopic Evaluation of the Degree of Graft Rejection

In order to microscopically assess the degree of graft rejection, histopathological analyses were made of tissue specimens obtained by resection of a piece of skin at a site showing no macroscopic signs of rejection to enable assessment of all the layers of the grafted skin and the periphery of the graft at the point of contact between the graft and the skin of the receiver. The sections were assessed using a scale based on the Banff 2007 pathomorphological classification (Table 2) (12). The presented scale allows analysis of both allo- and xenografts of skin. The analysis covered lymphocytic infiltrates in the dermis, around the blood vessels, and in the epidermis and appendages as well as the condition of the epidermis for the occurrence of apoptosis, dyskeratosis, and necrosis. The number of inflammatory cells in the graft was analyzed in two randomly selected fields with an area of 100 μm^2^. In addition, the presence of fibrosis in the region of the graft was assessed, but this did not affect the classification of the degree of graft rejection.

**Table 2:** Scale of histologic stages of the skin allo- and xenografts rejection.

**Grade**	**Description**	**Histology**	
		**Allografts**	**Xenografts**
0	Up to 10 lymphocytes/10^4^ μm^2^		
		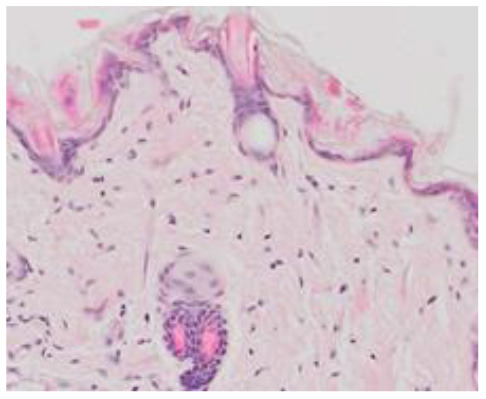	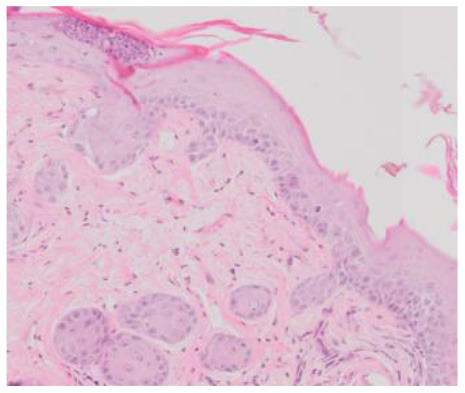
I	Perivascular infiltrations up to 20 lymphocytes/10^4^ μm^2^–with intact epidermis		
		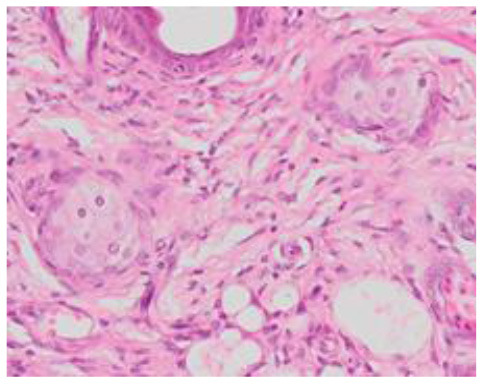	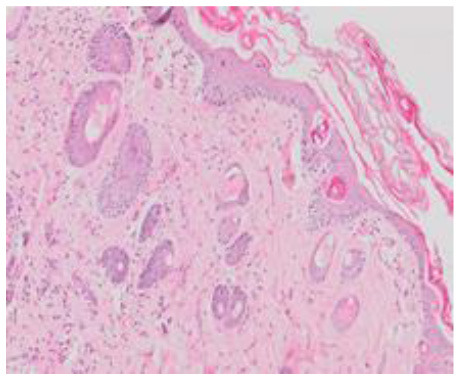
II	Perivascular infiltrations up to 30 lymphocytes/10^4^ μm^2^, presence of lymphocytes in the dermis, without apoptosis or necrosis in dermis		
		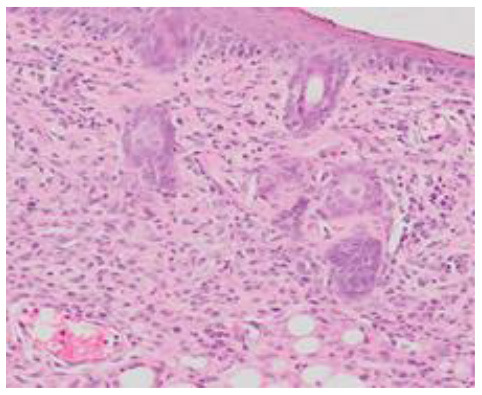	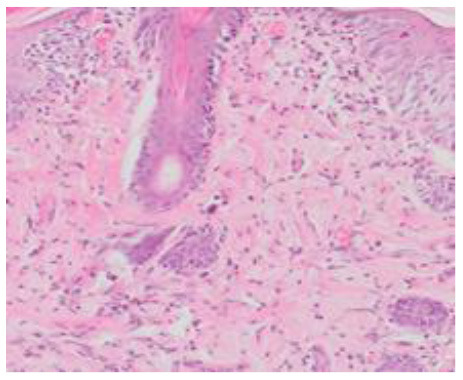
III	Dermal lymphocytic infiltrations more than 30 lymphocytes/10^4^ μm^2^ with dyskeratosis or apoptosis in epidermis		
		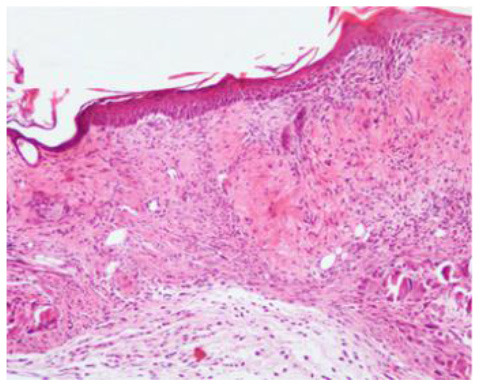	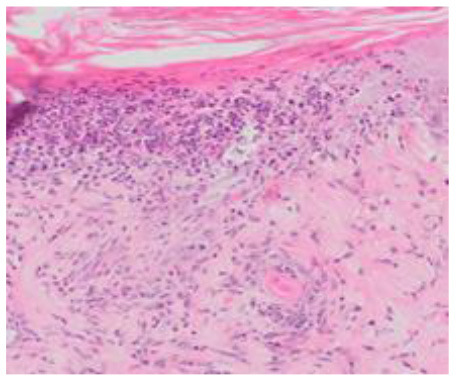
IV	Skin graft necrosis		
		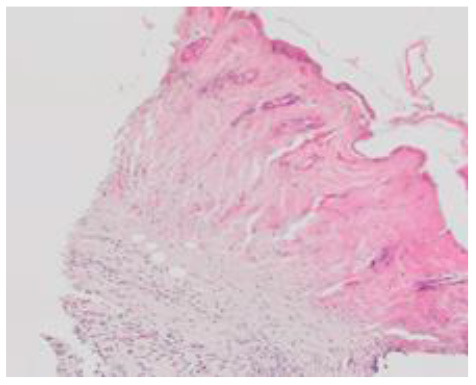	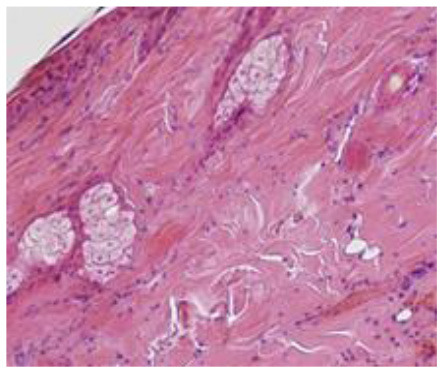

*This scale is based on the Banff Pathomorphological Classification 2007 (13), and includes the number of inflammatory cells in the graft. The images are representative of the individual scale steps (H-E staining, magn. 200x)*.

### Statistical Analysis

Statistical analyses were performed using Statistica 13 software. A chi-squared test was performed to select an appropriate test. For normal distribution, ANOVA test was used and, if necessary, also NIR and RIR Tukey *post-hoc* comparison tests for unequal counts. For non-normal distributions, U Mann–Whitney and Kruskal-Wallis tests were used. For independent groups, Student's *t*-test was also used in justified cases. The survival curves (Kaplan-Meier) of the grafts were compared between the groups using the log-rank test. The observed differences were considered to be statistically important at *p* < 0.05.

The data from the RT-PCR reactions were analyzed using RT^2^ Profiler PCR Array Data Analysis Template v. 3.5 software provided by the manufacturer of the kit (http://www.qiagen.com; SABiosciences, Qiagen).

## Results

### Characterization of hAC

The number of hAC cells remained at a similar level during 96-h cell culture. The hAC vitality was 88% and slightly decreased to 83% throughout the culture. At 12 h the majority of isolated cells (80%) expressed the pluripotency marker SSEA4 and more than half (53%) expressed the epithelial cell marker CK7. During the cultivation period, the number of hAC CK7+ and SSEA4+ remained almost constant, while the number of hAC HLA-G+ increased twice at 96 h. Also, the expression of the tested genes increased: HLA-G more than twelve fold, NOS2 almost two fold, and PTGS2 more than seven fold. In the conditioned media collected during the cultivation of activated hAC, an increase in the concentration of PGE2 (1.6x) and HLA-G (threefold) was determined at 96 h (Table 3). These results showed that after 96 h, hAC used in this experiment became activated in terms of their immunomodulatory properties in similar manner as it was described earlier (10).

**Table 3:** Quantitative and qualitative analysis of non-activated and cytokine-activated hAC after 96 h cell culture.

**Parameter**	**Value**	
Placenta weight [g]	482	
Placenta size [cm]	20 × 24	
Amnion mass [g]	9	
Total amnion cell count [mln]	123	
Isolation efficiency [mln/g]	13,7	
	**12 h**	**96 h**
Cell count [mln/bottle]	4	4.2
Vitality [MPI]	0.88	0.83
CK7^+^ cells [%]	53	52
SSEA4^+^ cells [%]	80	83
HLA-G^+^ cells [%]	18	35
*NOS2* expression	1	×1.85
*HLA-G* expression	1	×12.16
*PTGS2* expression	1	×7.5
HLA-G concentration [UI/10^6^ cells)	12	38
PGE2 concentration [pg/10^6^ cells)	380	620

### Comparison of the Immunosuppressive Efficacy of Activated Amniotic Cells and CsA After Skin Allo- and Xenotransplantation

**Figure 2:**
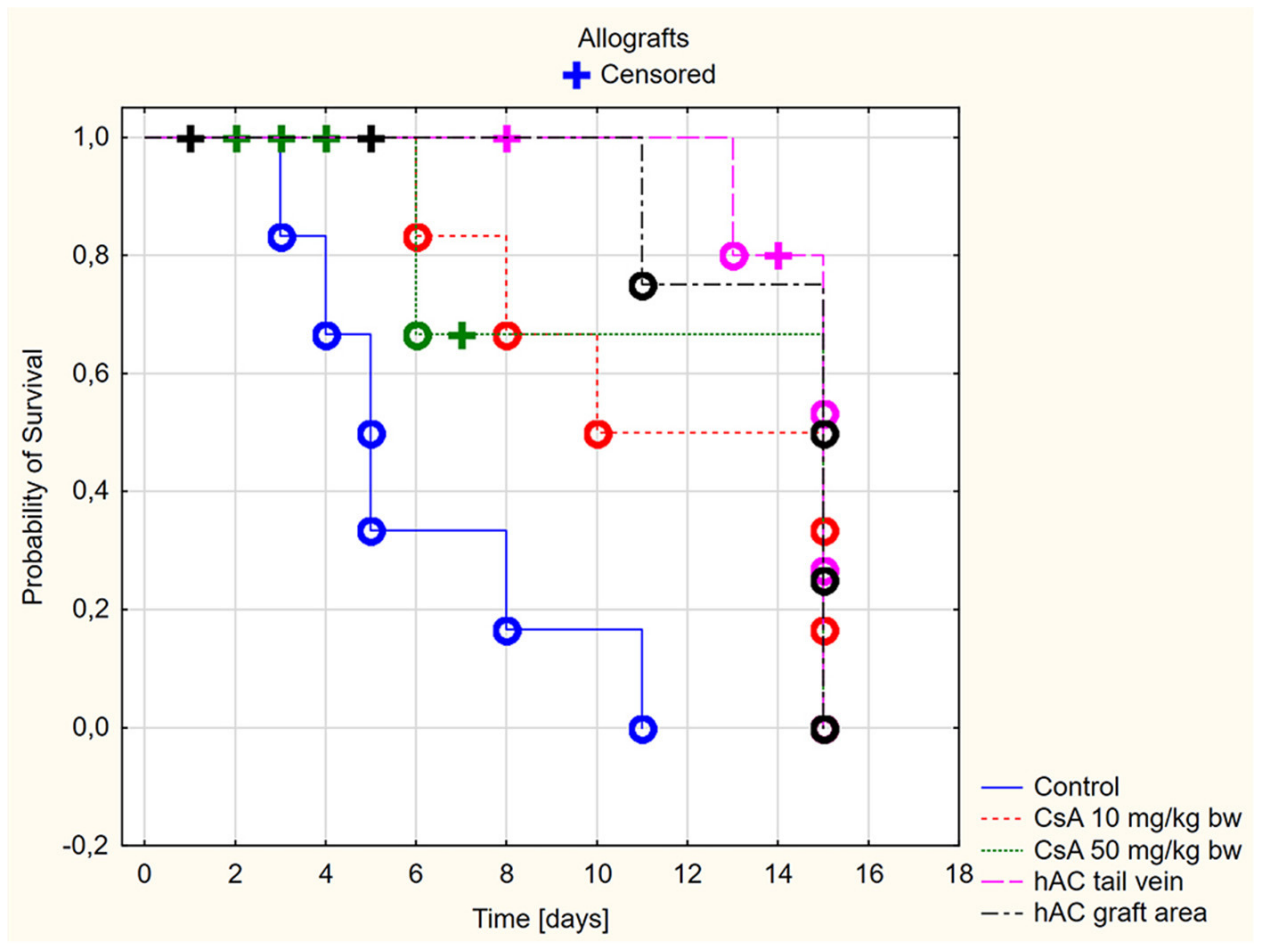
Skin allografts survival time. Censored (+)—individuals that left the study before the day of massive graft rejection or on 15th day of breeding, due to severe side effects; Completed (o)—individuals observed until 15th day of breeding or until the graft massive rejection has occurred (80% of the graft surface necrosis).

Based on the macroscopic evaluation of the grafts (Supplementary Table 1), during the 15-day experiment it was shown that in the groups of allografts receiving CsA at doses of 10 mg/kg bw and 50 mg/kg bw, the survival time of the grafts was significantly longer than that of the control group (*p* < 0.05, log-rank test). There was no statistically significant difference in graft survival between the groups receiving CsA at doses of 10 and 50 mg/kg bw (*p* = 0.87, log-rank test) (Figure 2).

However, more adverse reactions were observed in mice receiving the dose of 50 mg/kg bw. In the group of animals after allotransplantation, four mice were excluded from the experiment due to complications related to the use of a high dose of CsA (50 mg/kg bw). By day four from the time of transplantation, three recipient mice developed severe signs of CsA toxicity, such as loss of appetite, inactivity, and severe diarrhea. One mouse without any previous disturbing signs died on the eighth days of observation. In the group receiving CsA at the dose of 10 mg/kg bw, there were no signs indicating adverse effects of this substance.

In animals after skin allotransplantation receiving hAC to the tail vein or in the region of the graft, as in the group of animals receiving CsA at the dose of 10 mg/kg bw, the survival rate of the grafts was significantly longer compared to the control group (*p* < 0.05, log-rank test). No differences were found in the graft survival time in the groups receiving hAC—regardless of the route of cell administration—and CsA administered at the dose of 10 mg/kg bw (Figure 2). In animals administered with hAC, no adverse effects were observed due to cell administration.

**Figure 3:**
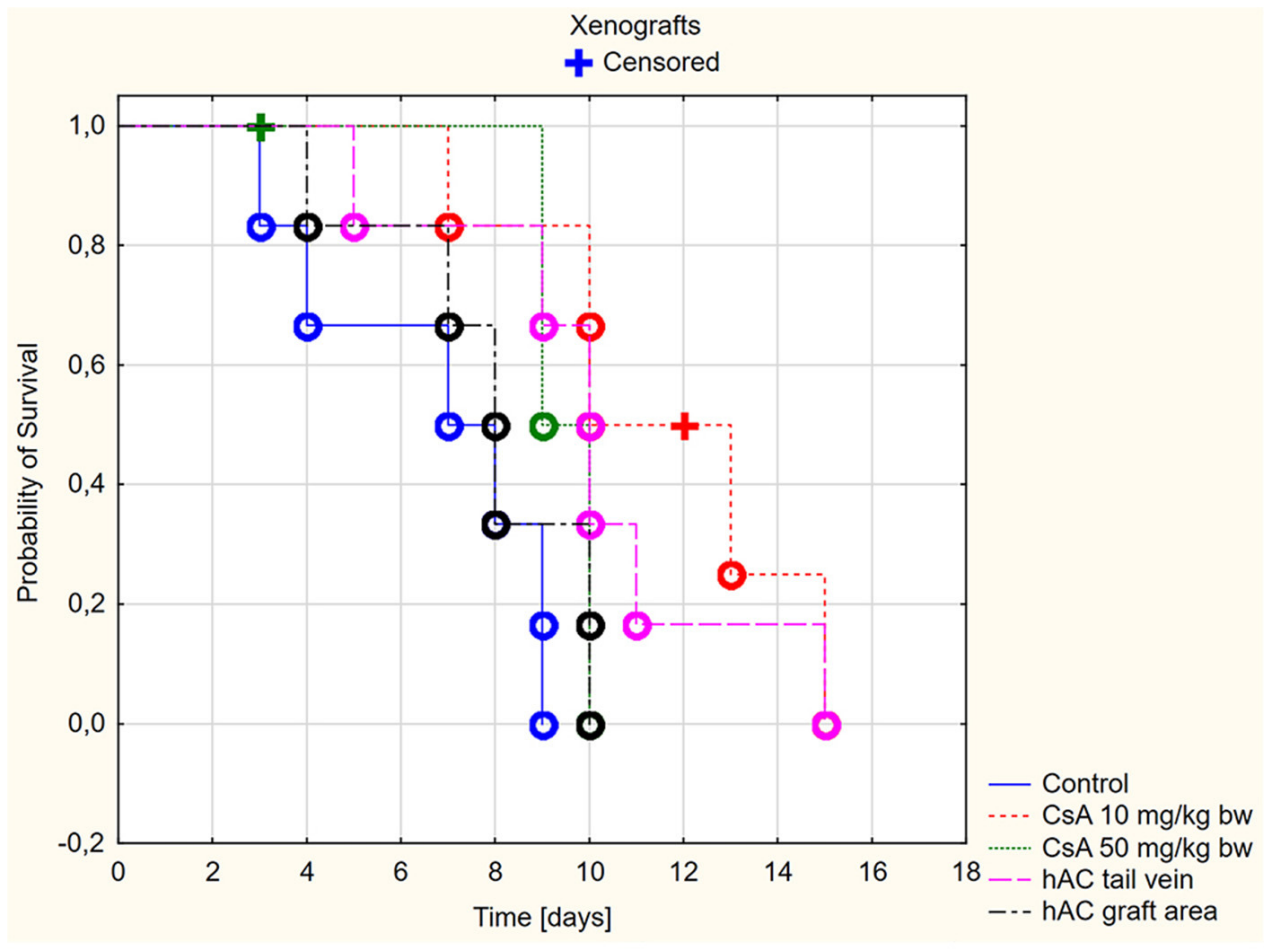
Skin xenografts survival time. Censored (+)—individuals that left the study before the day of massive graft rejection or on 15th day of breeding, due to severe side effects; Completed (o)—individuals observed until 15th day of breeding or until the graft massive rejection has occurred (80% of the graft surface necrosis).

In the xenograft group (Figure 3), the survival time of the grafts was significantly longer in the recipient mice receiving CsA at the dose of 10 mg/kg bw compared to the control group (*p* < 0.05, log-rank test). On the other hand, there was no difference in the graft survival time between the control group and the group receiving CsA at the dose of 50 mg/kg bw (*p* = 0.19, log-rank test).

Among xenografts, the dose of CsA 50 mg/kg bw also proved to be very toxic. Four mice were excluded from the group of animals receiving this dose due to severe organ complications. This number was comparable to the group of animals subjected to allotransplantation. The day after transplantation, they developed clinical signs such as lack of appetite, diarrhea, and decreased activity during wakefulness. Despite the low intensity of the described clinical signs, one mouse died on the second day of observation and the remaining animals died on the third day.

A much better safety profile was observed in the group receiving CsA at the dose of 10 mg/kg bw. Only one adverse event was reported: on day 12 of observation, one mouse died without any previous disturbing signs.

In animals after skin xenotransplantation in the group receiving hAC to the tail vein, the graft survival time was not significantly different (*p* < 0.05, log-rank test) compared to the group of animals receiving CsA at the dose of 10 mg/kg bw; nevertheless, it was significantly longer in both groups compared to the control group (*p* < 0.05, log-rank test). By contrast, in animals receiving hAC in the region of the graft, no difference was found in the graft survival time compared to the control group and the group administered with CsA at the dose of 50 mg/kg bw (Figure 3). As with the allograft group, no adverse effects were observed in connection with HAC administration.

### Microscopic Evaluation of Graft Survival in Mice Administered With hAC

After analyzing histological images of the grafts to assess the sizes of immune cell infiltrates and the condition of the epidermis, the point values were determined for the degree of graft rejection of each mouse (Supplementary Table 2).

At the end of the experiment, the degree of graft rejection at the microscopic level in the control group averaged III in both allo- and xenograft groups (Table 4). In the allograft group of mice receiving hAC, regardless of the site of administration and comparably to the group receiving CsA, the observed graft rejection rate remained on average at level I. In the xenograft group, in mice receiving hAC in the region of the graft, a higher average rejection rate (III) was observed compared to the group receiving hAC to the tail vein and CsA at the dose of 10 mg/kg bw (stage II) (Table 4).

**Table 4:** Percentage of individuals representing a specific stage of graft rejection (0–IV) in all studied groups of mice.

**Grade**	**Control**	**CsA10 mg/kg bw**	**CsA 50 mg/kg bw**	**hACtail vein inj**.	**hAC graft region inj**.
**Allografts**					
0	0%	16%	0%	16%	16%
I	0%	50%	50%	50%	68%
II	16%	34%	0%	34%	16%
III	68%	0%	50%	0%	0%
IV	16%	0%	0%	0%	0%
**Xenografts**					
0	0%	0%	0%	0%	0%
I	0%	40%	0%	0%	0%
II	0%	40%	50%	50%	34%
III	84%	20%	50%	50%	50%
IV	16%	0%	0%	0%	16%

The obtained results show that animals with transplanted allografts that received active hAC (regardless of the route of administration), as in the group receiving CsA, experienced significantly less inflammatory infiltration in the transplanted tissue compared to the control group (Table 4). In almost all animals after xenotransplantation, the immune response observed in the grafts and degradation of the epidermis were more intense than in the case of allografts. In the group of mice receiving hAC to the tail vein, inflammatory infiltrates in the grafts were significantly smaller than in the control group (*p* < 0.05) and similar to the infiltrates in the group of animals receiving CsA (*p* > 0.05). Administration of hAC in the xenograft region did not reduce the inflammatory infiltrate in the graft or inhibit epidermal degenerative changes compared to the control group. The observed increase in the inflammatory process in microscopic images was consistent with the macroscopic observations.

### Assessment of Inflammation in Recipient Mice

**Figure 4:**
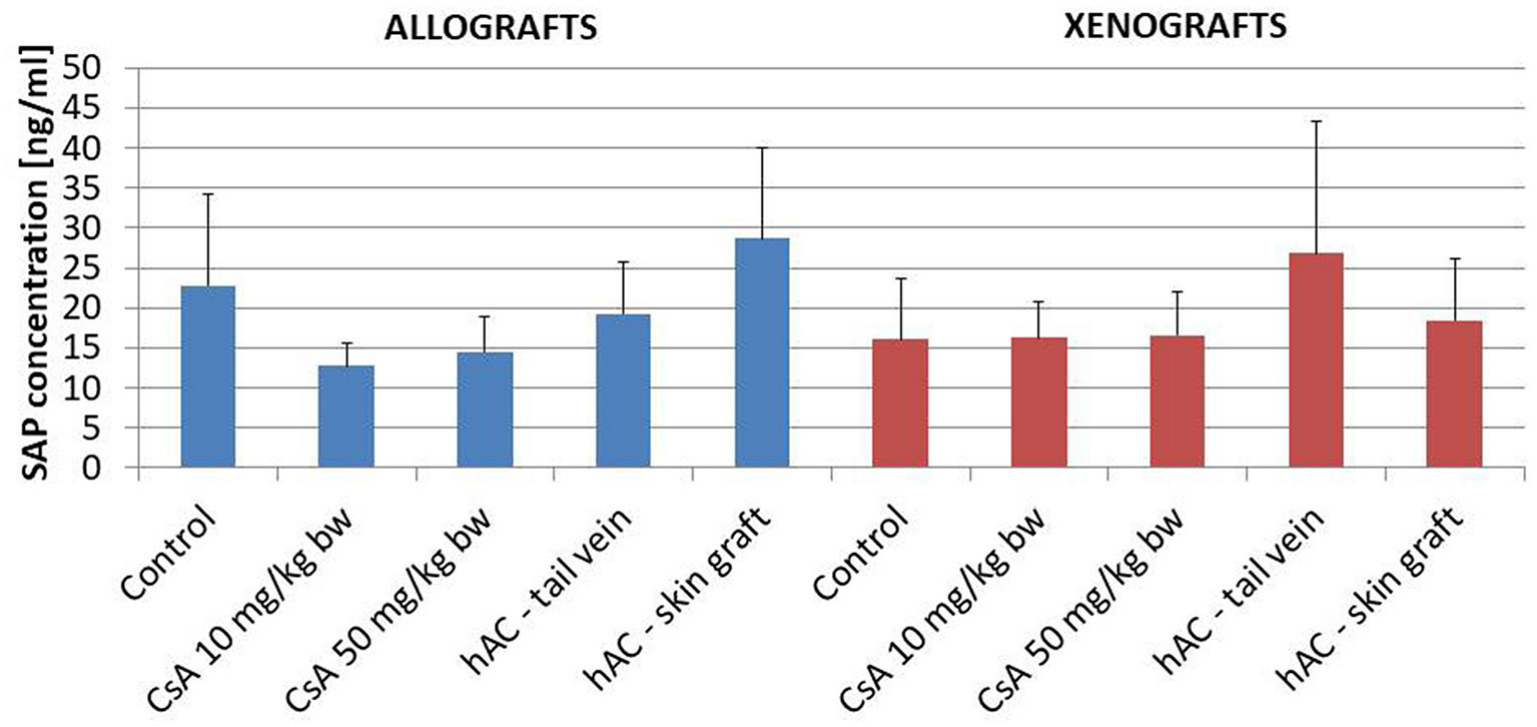
The results of Schilling test expressing the percentage of leukocytes in the mouse blood of studied groups. Blood smears were performed on the last day of the observation for each animal. The average values are presented in the graph; *n* = 10.

Blood smears were performed and the Schilling differential blood count was determined to assess the inflammatory response of the recipient mice. There were no statistically significant differences between the experimental groups and compared to the control groups (after administration of placebo and after administration of placebo and transplantation, which demonstrates a lack of systemic inflammatory response (Figure 4).

**Figure 5:**
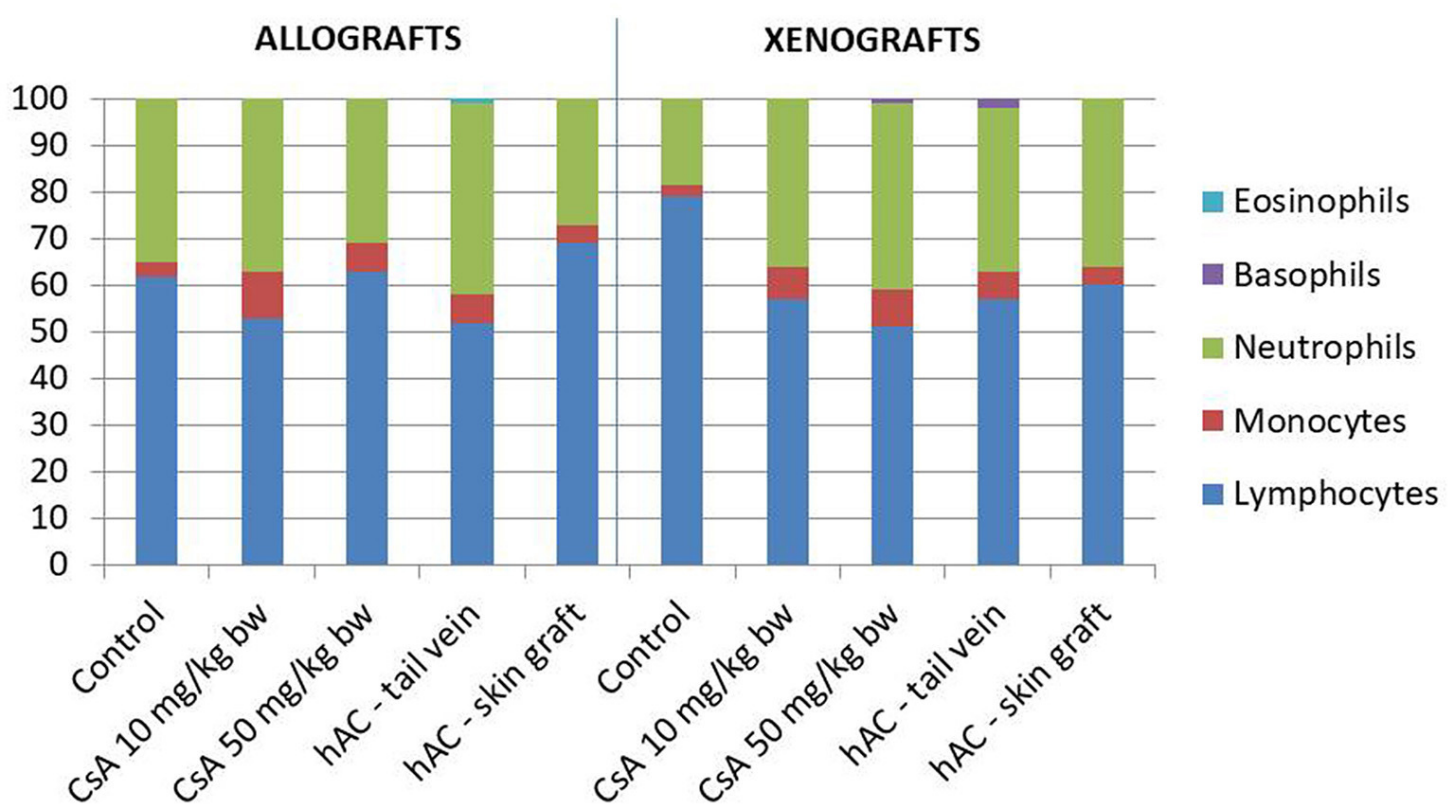
The concentration of the mouse acute phase marker S-amyloid P (SAP) in the serum of the recipient mice in the study groups on the last day of observation for the given animal; *n* = 10.

The lack of a systemic inflammatory response of the recipient was confirmed by testing the concentration of the murine SAP marker of the acute-phase response. In addition, this analysis found no statistically significant differences between the groups of animals, also in relation to the group of animals whose blood was collected for pre-transplantation testing (Figure 5).

## Discussion

Human amniotic cells perform important physiological functions related to, among others, the generation of maternal–fetal tolerance. Thanks to their immunomodulatory properties, they could be used in regenerative medicine and transplantology to inhibit the body's immune response against a tissue graft after allo- or even xenotransplantation. Moreover, both mesenchymal and epithelial amniotic cells lack of cell surface histocompatibility complex (HLA) class II and T cell costimulatory molecules, rendering them immunoprivileged. These characteristics allowed to deliver hAC across the immunologic barriers of allo- and xenogeneic transplant recipients, without pharmacological immunosuppression (14, 15). This makes it possible to carry out pre-clinical animal experiments creating conditions more closely related to the human transplantations. hAC have been successfully administered in animal models many times. For example, hAC alleviated and reversed the progression of bleomycin-induced lung fibrosis in mice (16). In chemotherapy-induced premature ovarian failure, hAC migrated to the injured ovaries and differentiated into granulosa cells to restore folliculogenesis and ovarian function (17). In myocardial infarction, hAC improved cardiac function (18). Moreover, there are some registered clinical trials where allogenic hAC have been used with successes with the maintenance of a good safety profile (ACTRN12616000437460, ACTRN12618000920291, ACTRN12618000076279) (19). The use of hAC as a safe and effective immunomodulatory agent seems to be a very promising alternative to the conventional pharmacological immunosuppression since the use of drugs such as CsA entails the risk of adverse effects.

Nonetheless, some studies point to potential limitations of hAC transplantations. It was reported that in some cases hAC can show immunogenic and stimulatory activities. But there is still not enough data to assess which agents are responsible for maintaining the balance between immunosuppressive and immunostimulatory properties or its shift into one of two sides (20). It is well known that INFγ stimulation augments the expression of HLA-ABC and CD40 in hAC what potentially reduces immunoprivilege of these cells, but also increases the expression of HLA-G causing an adverse effect (21). In addition, some reports indicate that after the infusion of hAC only few cells can be localized (20). However, in animal model of the skin transplantation, hAC infusion have been shown to develop long term effects, more than 50 days, especially when used with additional agents, namely CD4 and CD8 antibodies and a low dose of busulfan (22). It suggests that hAC injection can be more beneficial as an additional agent, not a single immunosuppressive factor.

The experiments carried out so far with the use of native hAC have not given a clear answer whether these cells are capable of inhibiting rejection of skin grafts after administration to the recipient (22). On the other hand, it has been shown that hAC need activation to significantly increase their immunomodulatory potential. So far it has been shown that after 96 h of culture, there is an increase in the expression of non-classical HLA-G histocompatibility antigen and the *NOS2, PTGS2*, and *HLA-G* genes—factors important during the acceptance of skin grafts and development of immunotolerance (10, 23, 24).

Despite previous studies on skin allo- and xenotransplantation models, which used different doses of CsA to induce tolerance to skin transplantation, it is generally recommended to determine in each case the effectiveness of a given CsA dose in the currently designed experiment (25). This is associated with the high species and even interstrain variability in the rate of CsA metabolism, which occurs mainly in the liver and is mediated by cytochrome P450IIIA (26). For that reason, in the first stage of the presented experiment, it was necessary to select the optimal dose of CsA, which would be both safe and effective.

The experiments conducted so far have shown that doses below 10 mg/kg bw can significantly increase the process of graft rejection. In a murine model of skin allotransplantation where such CsA doses were administered, a reduction in graft survival to a maximum of five days was observed, in contrast to mice receiving placebo, in which graft survival lasted up to 14 days in individual cases (7). The observed phenomenon is related to the inhibition of regulatory T (Treg) cells (27), whose activity is linked to the development of immune tolerance to the graft (28), by the administration of low doses of CsA.

In experiments performed so far, there have been no suppressive effect on Treg cells when using CsA doses higher than 10 mg/kg bw (27). When CsA was used in doses ranging from 10 to 25 mg/kg bw, the graft survival varied depending on the model of human skin xenotransplantation from several days in the case of full-thickness skin pieces (29) to even more than 100 days in the case of xenotransplantation of non-full-thickness skin pieces (5). In murine skin allotransplantation, graft survival was typically longer than 20 days (30). Admittedly, the use of CsA at the dose of 25 mg/kg bw is often accompanied by liver and kidney dysfunctions, resulting in abnormal levels of functional markers of these organs, for instance an increase in the level of transaminases or urea in blood serum, but these changes are reversible and do not affect the course of the experiment (5). Moreover, it has been shown that the higher the doses of CsA used in the range of 10–50 mg/kg bw, increase the tolerance to transplantation and decrease the infiltrates of lymphoid cells in the graft (12, 31, 32). It is suggested that the dose of 50 mg/kg bw should still be safe for animals (33) since only at doses above 50 mg/kg bw there is a significant increase in drug-related complications such as sever renal, hepatic, and cardiovascular failure, leading to rapid death of the animal (5, 17, 18, 34). Severe damage to organ functions leading to rapid animal death was usually observed only at doses higher than 50 mg/kg bw, such as 75 mg/kg bw or even 100 mg/kg bw (33).

Therefore, based on the observations made during previously published experiments in murine models, it could be assumed that the doses that are both safe and allow development of tolerance of the immune system of the mouse recipient to skin transplantation fall in the range of 10 do 50 mg/kg bw. The observation time in this experiment was set at 15 days, which is the minimum time required to obtain significant differences between placebo-treated animals and animals receiving immunosuppressive medication, achieving effective immunosuppression (30).

The analysis of efficiency of the CsA dose of 10 mg/kg bw conducted in this experiment showed that it significantly prolongs skin graft survival both in the allograft and xenograft groups compared to the control group. Macroscopic observations were supplemented with microscopic observations, which showed that in most preparations among allo- and xenografts the transplanted skin was characterized by normal histological structure with live epidermis and intact blood vessels. In the reticular layer of skin, small areas of fibrosis were observed in both allo- and xenografts. Similar histological changes were previously observed in the grafts in a rat model of human skin xenotransplantation (5), except that the lesions in the graft epidermis visible for more than 15 days also included to a much greater extent acanthosis of the epidermis and the presence of a large number of apoptotic cells.

In the presented experiment, a partial difference was demonstrated in the efficacy of both applied doses of CsA. Although the allograft mice administered with the CsA dose of 50 mg/kg bw, similarly to those administered with the CsA dose of 10 mg/kg bw, showed significantly longer survival of skin grafts compared to the control group, no such difference was found in the xenograft mice. Moreover, the histological preparations of skin specimens of both allo- and xenografts showed greater lymphocytic infiltrates in the group of mice receiving the CsA dose of 50 mg/kg bw compared to the lower dose. Following the administration of the CsA dose of 50 mg/kg bw, numerous complications were noted in the recipient mice with such signs as hematuria, lack of appetite, inactivity of the mice, or even sudden animal death. Due to these complications, eight mice (67%) were excluded from the experiment, which was seven animals more than in the group receiving CsA at the dose of 10 mg/kg bw. The observed signs suggested severe organ complications after the applied CsA dose, most likely related to liver and kidney damage. Moreover, despite maintaining sterile conditions during skin transplantation and the use of sterile dressings, it is not possible to exclude local inflammation due to graft infection. This may be related to a reduction of the defense capabilities of the body during immunosuppression.

In animals that were not excluded from the experiment, we examined the severity of the acute phase response induced by the use of selected doses of CsA. To this end, determinations were made of SAP protein concentrations (35) in the blood serum of recipient mice on the day of completion of skin graft observation. The analysis also covered the white blood cell differential count, which changes most often in the case of an ongoing inflammatory process (35, 36). Regardless of the study group, there were no significant changes in SAP concentration or in the percentage of white blood cells, which indicated the absence of a generalized inflammatory process and a low degree of organ damage in these animals.

To sum up, our analysis of the safety profile and efficacy of both CsA doses used in the presented experimental system showed that the dose of 10 mg/kg bw is both safe and effective. The use of the dose of 50 mg/kg bw leads to severe organ complications.

The experiment used hAC activated in a 96 h culture to develop graft tolerance in a model of skin allo- and xenotransplantation. After completion of macroscopic analyses of the degree of skin graft rejection supported by microscopic observations—in contrast to the results of experiments using native hAC described in the literature (22)—no statistically significant differences were found in the survival time of allografts in the groups receiving activated hAC or CsA at the dose of 10 mg/kg bw. It follows that activated hAC have an immunosuppressive potential similar to CsA. Somewhat different results were obtained in the group of mice after skin xenotransplantation. The difference in relation to the allograft group was noticeable in animals receiving hAC injections in the region of the graft, in whose case the graft survival time was significantly shorter and similar to the control group. Better immunosuppressive effects in this group might require an increase in the number of the subcutaneously administered cells due to the fact that a xenograft causes a stronger inflammatory response than an allograft. The different effects of hAC applied in the graft region in both groups could also be related to a slightly different immunomodulatory mechanism of hAC administered locally compared to the cells administered intravenously. A study using MSC in a murine model of myocardial infarction showed that cells administered intravenously, while passing through the pulmonary vessels, formed therein micromeboli (13, 14, 22, 37, 38). In these conglomerates, the action of MSC was enhanced by locally acting cytokines, which increased the release of, among others, PGE_2_, NO, TGS-6, and other cytokines into the bloodstream of the animal and positively affected the damaged myocardial tissue (38). Obviously, the mechanism described above may be one of many ways in which transplanted stem cells act globally (15).

The confirmed safety of use of hAC is also extremely important. So far, no evidence has been found that administration of these cells—when given in an optimal number and correct manner—causes adverse effects in the body of the recipient, such as formation of tumors or induction on an inflammatory process (15). Also, based on this study it can be concluded that the use of hAC is safe. No adverse effects were observed in mice receiving hAC, including loss of appetite, restlessness, and inactivity. There were no deaths among mice in all groups receiving hAC. In addition, the tested inflammatory markers—SAP level and Schilling differential blood count—were normal in these mice.

The animal model of skin allo- and xenotransplantation used in this study may be useful in designing experimental models for examining the response of rejection of human skin xenografts, investigating the properties of skin stem cells and skin diseases (e.g., pathophysiology of mild and severe dermatoses), and studying the effects of drugs and toxins on the skin. However before such model can be implemented, many difficulties need to be solved. Xenotransplantations are connected with very strong immunological reactions, and immunotolerance can be hardly assessed. A potential reaction occurring during or after human to mouse discordant xenotransplantation is the hyperacute rejection. Hyperacute rejection is connected with the presence of natural xenoreactive antibodies against discordant antigens on the vascular endothelium of the donor organ, which results in activation of the classical pathway of complement. To inhibit the hyperacute rejection mixed hematopoietic cell chimerism can be implemented, however it influences the animal model and produces difficulties in its clinical translation (39). Previously, discordant human skin transplantations were done with success with the use of nude or other immunodeficient mice (21). Admittedly, in these models it is possible to achieve transplant tolerance but also immunodeficient mice models have some limitations. There is impossible to assess the regular immunological interactions. Thus, in this study, we introduced an intermediate solution—xenotransplantation model with fully working immune system and without the risk of hyperacute rejection of the skin. It is well known that rat to mouse transplantations are concordant. It means that in recipient organism, immune reactions are similar to allotransplantation, but stronger and more rapid. The latter makes them as similar to discordant xenotransplantation but without hyperacute rejection (39). What is more, if we develop animal model of human to mouse skin transplantation the immunomodulatory effect of hAC might be too weak to keep the transplant alive so in this case hAC might play only supporting role to classical immunosuppression. The factor that can be helpful in relevance to skin allo- and xenotransplantation is Sirtuin 1 which is involved with intrinsic and extrinsic aging. The role of Sirtuin 1 activators may be critical to the success of the human skin xenografts and the role of Sirtuin 1 inhibitors may be critical to regenerative medicine and to presented animal model (40). Further analyses need to be done.

In conclusion, this experiment showed that hAC activated *ex vivo* by INFγ and IL-1β for 96 h and then administered to the recipient of a skin allo- or xenograft have the ability to inhibit graft rejection to a degree similar to a conventional immunosuppressant, CsA, administered at the dose of 10 mg/kg bw. However, it was shown that the route of administration of the cells transplanted to the body of the recipient is an important consideration for the final effect of such procedure. Neither subcutaneous nor intravenous routes of hAC administration significantly affect the end result of suppression of skin graft rejection in allotransplantation, but only intravenous administration is effective in xenotransplantation.

## Data Availability Statement

The original contributions presented in the study are included in the article/Supplementary Material, further inquiries can be directed to the corresponding author/s.

## Ethics Statement

The studies involving human participants were reviewed and approved by The Bioethics Committee of the Medical University of Silesia (SUM) in Katowice (decision no KNW/0022/KB/229/14). The patients/participants provided their written informed consent to participate in this study. The animal study was reviewed and approved by Local Ethical Committee on Animal Experiments (decision no 121/2015) of the SUM in Katowice.

## Author Contributions

EK: concept, review of literature, *in vitro* study, animal experimental models, data collection and interpretation, and writing original draft. AG: animal experimental models. PC: concept, design, review of literature, histological studies, data collection and interpretation, writing original draft, and manuscript review and editing. All authors contributed to the article and approved the submitted version.

## Funding

This work was supported by National Science Centre, grant PRELUDIUM no 2016/21/N/NZ6/02335, and SUM in Katowice, institutional grants no KNW-2-005/D/7/N and KNW-2-029/N/8/N.

## Conflict of Interest

The authors declare that the research was conducted in the absence of any commercial or financial relationships that could be construed as a potential conflict of interest.

## Publisher's Note

All claims expressed in this article are solely those of the authors and do not necessarily represent those of their affiliated organizations, or those of the publisher, the editors and the reviewers. Any product that may be evaluated in this article, or claim that may be made by its manufacturer, is not guaranteed or endorsed by the publisher.
