# Supplementary material for: Immunosuppressive Potential of Activated Human Amniotic Cells in an Experimental Murine Model of Skin Allo- and Xenotransplantation
Source: Front Med (Lausanne). 2021 Sep 23;8:715590. doi: 10.3389/fmed.2021.715590 (PMC8494785; doi:10.3389/fmed.2021.715590)
Supplement: Supplementary file 1 [file Table_1.DOCX]

Supplementary Material

# Supplementary Tables

**Supplementary Table 1.** The macroscopic assessment of the skin graft rejection according to the scale applied to this study (Table 1). X – the end of the observation due to graft necrosis..

| Control – allografts | | | | | | | | | | | | | | | | | | | | | | | | | | | | | | |
| --- | --- | --- | --- | --- | --- | --- | --- | --- | --- | --- | --- | --- | --- | --- | --- | --- | --- | --- | --- | --- | --- | --- | --- | --- | --- | --- | --- | --- | --- | --- |
|  |  | |  | |  | |  |  | |  | |  | |  | |  | |  | |  | |  | | |  | |  | |  | |
|  | Day 1 | Day 2 | | Day 3 | | Day 4 | | | Day 5 | | Day 6 | | Day 7 | | Day 8 | | Day 9 | | Day 10 | | Day 11 | | Day 12 | Day 13 | | Day 14 | | Day 15 | |  |
| Mouse 1 | 0 | 0 | | 4 | |  | | |  | |  | |  | |  | |  | |  | |  | |  |  | |  | |  | |  |
| Mouse 2 | 0 | 0 | | 3 | | 4 | | |  | |  | |  | |  | |  | |  | |  | |  |  | |  | |  | |  |
| Mouse 3 | 0 | 0 | | 2 | | 3 | | | 4 | |  | |  | |  | |  | |  | |  | |  |  | |  | |  | |  |
| Mouse 4 | 0 | 0 | | 2 | | 3 | | | 4 | |  | |  | |  | |  | |  | |  | |  |  | |  | |  | |  |
| Mouse 5 | 0 | 0 | | 1 | | 2 | | | 2 | | 3 | | 3 | | 4 | |  | |  | |  | |  |  | |  | |  | |  |
| Mouse 6 | 0 | 0 | | 0 | | 1 | | | 2 | | 2 | | 2 | | 3 | | 3 | | 3 | | 4 | |  |  | |  | |  | |  |
|  |  |  | |  | |  | | |  | |  | |  | |  | |  | |  | |  | |  |  | |  | |  | |  |
| Control – xenografts | | | | | | | | | | | | | | | | | | | | | | | | | | | | | | |
|  |  |  | |  | |  | | |  | |  | |  | |  | |  | |  | |  | |  |  | |  | |  | |  |
|  | Day 1 | Day 2 | | Day 3 | | Day 4 | | | Day 5 | | Day 6 | | Day 7 | | Day 8 | | Day 9 | | Day 10 | | Day 11 | | Day 12 | Day 13 | | Day 14 | | Day 15 | |  |
| Mouse 1 | 0 | 0 | | 4 | |  | | |  | |  | |  | |  | |  | |  | |  | |  |  | |  | |  | |  |
| Mouse 2 | 0 | 0 | | 3 | | 4 | | |  | |  | |  | |  | |  | |  | |  | |  |  | |  | |  | |  |
| Mouse 3 | 0 | 0 | | 2 | | 3 | | | 3 | | 3 | | 4 | |  | |  | |  | |  | |  |  | |  | |  | |  |
| Mouse 4 | 0 | 0 | | 2 | | 2 | | | 2 | | 3 | | 3 | | 4 | |  | |  | |  | |  |  | |  | |  | |  |
| Mouse 5 | 0 | 0 | | 1 | | 2 | | | 2 | | 3 | | 3 | | 3 | | 4 | |  | |  | |  |  | |  | |  | |  |
| Mouse 6 | 0 | 0 | | 0 | | 1 | | | 2 | | 3 | | 3 | | 3 | | 4 | |  | |  | |  |  | |  | |  | |  |
|  |  |  | |  | |  | | |  | |  | |  | |  | |  | |  | |  | |  |  | |  | |  | |  |
| CsA 10 mg/kg bw – allografts | | | | | | | | | | | | | | | | | | | | | | | | | | | | | | |
|  |  |  | |  | |  | | |  | |  | |  | |  | |  | |  | |  | |  |  | |  | |  | |  |
|  | Day 1 | Day 2 | | Day 3 | | Day 4 | | | Day 5 | | Day 6 | | Day 7 | | Day 8 | | Day 9 | | Day 10 | | Day 11 | | Day 12 | Day 13 | | Day 14 | | Day 15 | |  |
| Mouse 1 | 0 | 2 | | 3 | | 3 | | | 3 | | 4 | |  | |  | |  | |  | |  | |  |  | |  | |  | |  |
| Mouse 2 | 0 | 0 | | 2 | | 2 | | | 2 | | 3 | | 3 | | 4 | |  | |  | |  | |  |  | |  | |  | |  |
| Mouse 3 | 0 | 0 | | 2 | | 2 | | | 2 | | 2 | | 2 | | 3 | | 3 | | 4 | |  | |  |  | |  | |  | |  |
| Mouse 4 | 0 | 0 | | 1 | | 1 | | | 1 | | 1 | | 2 | | 2 | | 2 | | 2 | | 2 | | 3 | 3 | | 3 | | 3 | |  |
| Mouse 5 | 0 | 0 | | 1 | | 2 | | | 2 | | 2 | | 2 | | 2 | | 2 | | 2 | | 2 | | 2 | 3 | | 3 | | 3 | |  |
| Mouse 6 | 0 | 0 | | 0 | | 0 | | | 1 | | 1 | | 1 | | 1 | | 1 | | 1 | | 2 | | 2 | 2 | | 2 | | 2 | |  |
|  |  |  | |  | |  | | |  | |  | |  | |  | |  | |  | |  | |  |  | |  | |  | |  |
| CsA 10 mg/kg bw – xenografts | | | | | | | | | | | | | | | | | | | | | | | | | | | | | | |
|  |  |  | |  | |  | | |  | |  | |  | |  | |  | |  | |  | |  |  | |  | |  | |  |
|  | Day 1 | Day 2 | | Day 3 | | Day 4 | | | Day 5 | | Day 6 | | Day 7 | | Day 8 | | Day 9 | | Day 10 | | Day 11 | | Day 12 | Day 13 | | Day 14 | | Day 15 | |  |
| Mouse 1 | 0 | 2 | | 3 | | 3 | | | 3 | | 3 | | 4 | |  | |  | |  | |  | |  |  | |  | |  | |  |
| Mouse 2 | 0 | 0 | | 2 | | 2 | | | 2 | | 3 | | 3 | | 3 | | 3 | | 4 | |  | |  |  | |  | |  | |  |
| Mouse 3 | 0 | 0 | | 2 | | 2 | | | 2 | | 2 | | 2 | | 3 | | 3 | | 4 | |  | |  |  | |  | |  | |  |
| Mouse 4 | 0 | 0 | | 1 | | 1 | | | 1 | | 1 | | 2 | | 2 | | 2 | | 2 | | 3 | | X |  | |  | |  | |  |
| Mouse 5 | 0 | 0 | | 1 | | 2 | | | 2 | | 2 | | 2 | | 2 | | 2 | | 2 | | 2 | | 2 | 4 | |  | |  | |  |
| Mouse 6 | 0 | 0 | | 0 | | 0 | | | 1 | | 1 | | 1 | | 1 | | 1 | | 1 | | 2 | | 2 | 2 | | 2 | | 3 | |  |
|  |  |  | |  | |  | | |  | |  | |  | |  | |  | |  | |  | |  |  | |  | |  | |  |
| CsA 50 mg/kg bw – allografts | | | | | | | | | | | | | | | | | | | | | | | | | | | | | | |
|  |  |  | |  | |  | | |  | |  | |  | |  | |  | |  | |  | |  |  | |  | |  | |  |
|  | Day 1 | Day 2 | | Day 3 | | Day 4 | | | Day 5 | | Day 6 | | Day 7 | | Day 8 | | Day 9 | | Day 10 | | Day 11 | | Day 12 | Day 13 | | Day 14 | | Day 15 | |  |
| Mouse 1 | 0 | 4 | |  | |  | | |  | |  | |  | |  | |  | |  | |  | |  |  | |  | |  | |  |
| Mouse 2 | 0 | 0 | | 4 | |  | | |  | |  | |  | |  | |  | |  | |  | |  |  | |  | |  | |  |
| Mouse 3 | 0 | 0 | | 2 | | 4 | | |  | |  | |  | |  | |  | |  | |  | |  |  | |  | |  | |  |
| Mouse 4 | 0 | 0 | | 1 | | 1 | | | 1 | | 4 | |  | |  | |  | |  | |  | |  |  | |  | |  | |  |
| Mouse 5 | 0 | 0 | | 1 | | 2 | | | 2 | | 2 | | 4 | |  | |  | |  | |  | |  |  | |  | |  | |  |
| Mouse 6 | 0 | 0 | | 0 | | 0 | | | 1 | | 1 | | 1 | | 1 | | 1 | | 1 | | 2 | | 2 | 2 | | 2 | | 3 | |  |
|  |  |  | |  | |  | | |  | |  | |  | |  | |  | |  | |  | |  |  | |  | |  | |  |
| CsA 50 mg/kg bw – xenografts | | | | | | | | | | | | | | | | | | | | | | | | | | | | | | |
|  |  |  | |  | |  | | |  | |  | |  | |  | |  | |  | |  | |  |  | |  | |  | |  |
|  | Day 1 | Day 2 | | Day 3 | | Day 4 | | | Day 5 | | Day 6 | | Day 7 | | Day 8 | | Day 9 | | Day 10 | | Day 11 | | Day 12 | Day 13 | | Day 14 | | Day 15 | |  |
| Mouse 1 | 0 | 2 | | X | |  | | |  | |  | |  | |  | |  | |  | |  | |  |  | |  | |  | |  |
| Mouse 2 | 0 | 0 | | X | |  | | |  | |  | |  | |  | |  | |  | |  | |  |  | |  | |  | |  |
| Mouse 3 | 0 | 0 | | X | |  | | |  | |  | |  | |  | |  | |  | |  | |  |  | |  | |  | |  |
| Mouse 4 | 0 | 0 | | X | |  | | |  | |  | |  | |  | |  | |  | |  | |  |  | |  | |  | |  |
| Mouse 5 | 0 | 0 | | 1 | | 2 | | | 2 | | 2 | | 2 | | 2 | | 4 | |  | |  | |  |  | |  | |  | |  |
| Mouse 6 | 0 | 0 | | 0 | | 0 | | | 1 | | 1 | | 1 | | 1 | | 1 | | 4 | |  | |  |  | |  | |  | |  |
|  |  |  | |  | |  | | |  | |  | |  | |  | |  | |  | |  | |  |  | |  | |  | |  |
| hAC tail vein inj. – allografts | | | | | | | | | | | | | | | | | | | | | | | | | | | | | | |
|  |  |  | |  | |  | | |  | |  | |  | |  | |  | |  | |  | |  |  | |  | |  | |  |
|  | Day 1 | Day 2 | | Day 3 | | Day 4 | | | Day 5 | | Day 6 | | Day 7 | | Day 8 | | Day 9 | | Day 10 | | Day 11 | | Day 12 | Day 13 | | Day 14 | | Day 15 | |  |
| Mouse 1 | 0 | 0 | | 1 | | 1 | | | 2 | | 2 | | 3 | | X | |  | |  | |  | |  |  | |  | |  | |  |
| Mouse 2 | 0 | 0 | | 1 | | 1 | | | 1 | | 1 | | 2 | | 2 | | 2 | | 2 | | 2 | | 2 | 4 | |  | |  | |  |
| Mouse 3 | 0 | 0 | | 2 | | 2 | | | 2 | | 2 | | 2 | | 2 | | 2 | | 2 | | 2 | | 2 | 3 | | X | |  | |  |
| Mouse 4 | 0 | 0 | | 1 | | 1 | | | 1 | | 1 | | 2 | | 2 | | 2 | | 2 | | 2 | | 2 | 2 | | 2 | | 2 | |  |
| Mouse 5 | 0 | 0 | | 1 | | 1 | | | 2 | | 2 | | 2 | | 2 | | 2 | | 2 | | 2 | | 2 | 2 | | 2 | | 2 | |  |
| Mouse 6 | 0 | 0 | | 0 | | 0 | | | 1 | | 1 | | 1 | | 1 | | 1 | | 1 | | 2 | | 2 | 3 | | 3 | | 3 | |  |
|  |  |  | |  | |  | | |  | |  | |  | |  | |  | |  | |  | |  |  | |  | |  | |  |
| hAC tail vein inj. – xenografts | | | | | | | | | | | | | | | | | | | | | | | | | | | | | | |
|  |  |  | |  | |  | | |  | |  | |  | |  | |  | |  | |  | |  |  | |  | |  | |  |
|  | Day 1 | Day 2 | | Day 3 | | Day 4 | | | Day 5 | | Day 6 | | Day 7 | | Day 8 | | Day 9 | | Day 10 | | Day 11 | | Day 12 | Day 13 | | Day 14 | | Day 15 | |  |
| Mouse 1 | 0 | 0 | | 1 | | 1 | | | 4 | |  | |  | |  | |  | |  | |  | |  |  | |  | |  | |  |
| Mouse 2 | 0 | 0 | | 1 | | 1 | | | 1 | | 1 | | 2 | | 2 | | 4 | |  | |  | |  |  | |  | |  | |  |
| Mouse 3 | 0 | 0 | | 2 | | 2 | | | 2 | | 2 | | 2 | | 2 | | 2 | | 4 | |  | |  |  | |  | |  | |  |
| Mouse 4 | 0 | 0 | | 1 | | 1 | | | 1 | | 1 | | 2 | | 2 | | 2 | | 4 | |  | |  |  | |  | |  | |  |
| Mouse 5 | 0 | 0 | | 1 | | 1 | | | 2 | | 2 | | 2 | | 2 | | 2 | | 2 | | 4 | |  |  | |  | |  | |  |
| Mouse 6 | 0 | 0 | | 0 | | 0 | | | 1 | | 1 | | 1 | | 1 | | 1 | | 1 | | 2 | | 2 | 3 | | 3 | | 3 | |  |
|  |  |  | |  | |  | | |  | |  | |  | |  | |  | |  | |  | |  |  | |  | |  | |  |
| hAC graft area inj. – allografts | | | | | | | | | | | | | | | | | | | | | | | | | | | | | | |
|  |  |  | |  | |  | | |  | |  | |  | |  | |  | |  | |  | |  |  | |  | |  | |  |
|  | Day 1 | Day 2 | | Day 3 | | Day 4 | | | Day 5 | | Day 6 | | Day 7 | | Day 8 | | Day 9 | | Day 10 | | Day 11 | | Day 12 | Day 13 | | Day 14 | | Day 15 | |  |
| Mouse 1 | X |  | |  | |  | | |  | |  | |  | |  | |  | |  | |  | |  |  | |  | |  | |  |
| Mouse 2 | 0 | 0 | | 0 | | 0 | | | X | |  | |  | |  | |  | |  | |  | |  |  | |  | |  | |  |
| Mouse 3 | 0 | 0 | | 0 | | 0 | | | 1 | | 2 | | 2 | | 2 | | 3 | | 3 | | 4 | |  |  | |  | |  | |  |
| Mouse 4 | 0 | 0 | | 0 | | 1 | | | 1 | | 1 | | 2 | | 2 | | 2 | | 2 | | 2 | | 2 | 2 | | 2 | | 2 | |  |
| Mouse 5 | 0 | 0 | | 0 | | 1 | | | 2 | | 2 | | 2 | | 2 | | 2 | | 2 | | 2 | | 2 | 2 | | 2 | | 2 | |  |
| Mouse 6 | 0 | 0 | | 0 | | 1 | | | 1 | | 1 | | 1 | | 1 | | 1 | | 1 | | 2 | | 2 | 3 | | 3 | | 3 | |  |
|  |  |  | |  | |  | | |  | |  | |  | |  | |  | |  | |  | |  |  | |  | |  | |  |
| hAC graft area inj. – xenografts | | | | | | | | | | | | | | | | | | | | | | | | | | | | | | |
|  |  |  | |  | |  | | |  | |  | |  | |  | |  | |  | |  | |  |  | |  | |  | |  |
|  | Day 1 | Day 2 | | Day 3 | | Day 4 | | | Day 5 | | Day 6 | | Day 7 | | Day 8 | | Day 9 | | Day 10 | | Day 11 | | Day 12 | Day 13 | | Day 14 | | Day 15 | |  |
| Mouse 1 | 0 | 0 | | 0 | | 4 | | |  | |  | |  | |  | |  | |  | |  | |  |  | |  | |  | |  |
| Mouse 2 | 0 | 0 | | 0 | | 0 | | | 2 | | 2 | | 4 | |  | |  | |  | |  | |  |  | |  | |  | |  |
| Mouse 3 | 0 | 0 | | 0 | | 0 | | | 3 | | 3 | | 3 | | 4 | |  | |  | |  | |  |  | |  | |  | |  |
| Mouse 4 | 0 | 0 | | 0 | | 1 | | | 1 | | 1 | | 2 | | 4 | |  | |  | |  | |  |  | |  | |  | |  |
| Mouse 5 | 0 | 0 | | 0 | | 1 | | | 2 | | 2 | | 3 | | 3 | | 3 | | 4 | |  | |  |  | |  | |  | |  |
| Mouse 6 | 0 | 0 | | 0 | | 1 | | | 2 | | 2 | | 3 | | 3 | | 3 | | 4 | |  | |  |  | |  | |  | |  |

**Supplementary Table 2.** Results of observations of individual mice during the experiment. The results of lymphocytic infiltrations are mean values of cell counts in two fields of 100 µm^2^. Magn. 200x. Individuals censored due to side effects are presented in grey.

| Mouse | Dermal lymphocytic infiltration  [lymphocytes/100µm^2^] | Perivascular lymphocytic infiltration  [lymphocytes/100µm^2^] | Dermal and/or dermal appandages lymphocytic infiltration  [lymphocytes/100µm^2^] | Apoptosis, dyskeratosis and/or epidermal keratynolysis | Skin graft necrosis  (*Frank necrosis*) | Grade |
| --- | --- | --- | --- | --- | --- | --- |
| Control – allografts | | | | | | |
| 1 | 50 | 30 | 30 | present | absent | III |
| 2 | 61 | 32 | 21 | present | absent | III |
| 3 | 74 | 50 | 10 | present | absent | III |
| 4 | 45 | 30 | 15 | present | absent | III |
| 5 | 2 | 25 | - | absent | absent | II |
| 6 | - | - | - | - | present | IV |
| Control – xenografts | | | | | | |
| 1 | 34 | 13 | 11 | present | absent | III |
| 2 | 16 | 32 | 13 | present | absent | III |
| 3 | 73 | 35 | 12 | present | absent | III |
| 4 | 18 | 34 | 12 | present | absent | III |
| 5 | 40 | 45 | 7 | present | absent | III |
| 6 | - | - | - | - | present | IV |
| CsA 10 mg/kg bw – allografts | | | | | | |
| 1 | 9 | 0 | 3 | absent | absent | 0 |
| 2 | 10 | 4 | 1 | absent | absent | I |
| 3 | 17 | 2 | 3 | absent | absent | I |
| 4 | 14 | 3 | 2 | absent | absent | I |
| 5 | 24 | 15 | 3 | absent | absent | II |
| 6 | 23 | 24 | 4 | present | absent | II |
| CsA 10 mg/kg bw – xenografts | | | | | | |
| 1 | 14 | 2 | 4 | absent | absent | I |
| 2 | 17 | 3 | 2 | absent | absent | I |
| 3 | 16 | 8 | 7 | absent | absent | I |
| 4 | 18 | 16 | 3 | absent | absent | II |
| 5 | 17 | 18 | 6 | present | absent | II |
| 6 | 40 | 44 | 9 | Present | nieobecna | III |
| CsA 50 mg/kg bw – allografts | | | | | | |
| 1 | 12 | 0 | 3 | absent | absent | 0 |
| 2 | 4 | 0 | 1 | absent | absent | 0 |
| 3 | 2 | 4 | 3 | absent | absent | 0 |
| 4 | 17 | 2 | 3 | absent | absent | I |
| 5 | 14 | 3 | 2 | absent | absent | I |
| 6 | 48 | 45 | 7 | present | absent | III |
| CsA 50 mg/kg bw – xenografts | | | | | | |
| 1 | 12 | 8 | 4 | present | absent | I |
| 2 | 14 | 7 | 2 | present | absent | I |
| 3 | 18 | 19 | 3 | absent | absent | II |
| 4 | 19 | 18 | 6 | present | absent | II |
| 5 | 17 | 19 | 5 | present | absent | II |
| 6 | 49 | 40 | 9 | present | absent | III |
| hAC tail vein inj. – allografts | | | | | | |
| 1 | 3 | 0 | 3 | absent | absent | 0 |
| 2 | 13 | 4 | 2 | absent | absent | I |
| 3 | 19 | 2 | 3 | absent | absent | I |
| 4 | 14 | 10 | 0 | absent | absent | I |
| 5 | 27 | 15 | 8 | absent | absent | II |
| 6 | 26 | 28 | 5 | present | absent | II |
| hAC tail vein inj. – xenografts | | | | | | |
| 1 | 15 | 16 | 8 | absent | absent | I |
| 2 | 19 | 18 | 3 | absent | absent | I |
| 3 | 18 | 10 | 4 | absent | absent | I |
| 4 | 16 | 4 | 8 | absent | absent | I |
| 5 | 28 | 32 | 3 | present | absent | II |
| 6 | 49 | 43 | 14 | present | absent | III |
| hAC graft region inj. – allografts | | | | | | |
| 1 | 3 | 0 | 3 | absent | absent | 0 |
| 2 | 10 | 8 | 1 | absent | absent | I |
| 3 | 19 | 8 | 3 | absent | absent | I |
| 4 | 14 | 10 | 3 | absent | absent | I |
| 5 | 16 | 4 | 8 | absent | absent | I |
| 6 | 28 | 25 | 3 | absent | absent | II |
| hAC graft region inj. – xenografts | | | | | | |
| 1 | 16 | 14 | 4 | absent | absent | II |
| 2 | 26 | 27 | 32 | present | absent | II |
| 3 | 34 | 43 | 28 | present | absent | III |
| 4 | 25 | 46 | 31 | present | absent | III |
| 5 | 64 | 38 | 25 | present | absent | III |
| 6 | - | - | - | - | present | IV |
